# Progressive Brain Structural Impairment Assessed *via* Network and Causal Analysis in Patients With Hepatitis B Virus-Related Cirrhosis

**DOI:** 10.3389/fneur.2022.849571

**Published:** 2022-05-06

**Authors:** Shiwei Lin, Zheng Guo, Shengli Chen, Xiaoshan Lin, Min Ye, Yingwei Qiu

**Affiliations:** ^1^Department of Radiology, Huazhong University of Science and Technology Union Shenzhen Hospital, Shenzhen, China; ^2^Department of Radiology, The Third Affiliated Hospital of Guangzhou Medical University, Guangzhou Medical University, Guangzhou, China; ^3^Department of Hematology and Oncology, International Cancer Center, Shenzhen Key Laboratory of Precision Medicine for Hematological Malignancies, Shenzhen University General Hospital, Shenzhen University Clinical Medical Academy, Shenzhen University Health Science Center, Shenzhen, China; ^4^Department of Geriatrics, Guangzhou First People's Hospital, School of Medicine, South China University of Technology, Guangzhou, China; ^5^Department of Geriatrics, Guangzhou First People's Hospital, Guangzhou Medical University, Guangzhou, China

**Keywords:** cirrhosis, hepatic encephalopathy, MRI, gray matter, thalamus

## Abstract

**Objectives:**

This research amid to elucidate the disease stage-specific spatial patterns and the probable sequences of gray matter (GM) deterioration as well as the causal relationship among structural network components in hepatitis B virus-related cirrhosis (HBV-RC) patients.

**Methods:**

Totally 30 HBV-RC patients and 38 healthy controls (HC) were recruited for this study. High-resolution T1-weighted magnetic resonance imaging and psychometric hepatic encephalopathy score (PHES) were evaluated in all participants. Voxel-based morphometry (VBM), structural covariance network (SCN), and causal SCN (CaSCN) were applied to identify the disease stage-specific GM abnormalities in morphology and network, as well as their causal relationship.

**Results:**

Compared to HC (0.443 ± 0.073 cm3), the thalamus swelled significantly in the no minimal hepatic encephalopathy (NMHE) stage (0.607 ± 0.154 cm3, *p* <0.05, corrected) and further progressed and expanded to the bilateral basal ganglia, the cortices, and the cerebellum in the MHE stage (*p* < 0.05, corrected). Furthermore, the thalamus swelling had a causal effect on other parts of cortex-basal ganglia-thalamus circuits (*p* < 0.05, corrected), which was negatively correlated with cognitive performance (*r* = −0.422, *p* < 0.05). Moreover, the thalamus-related SCN also displayed progressive deterioration as the disease advanced in HBV-RC patients (*p* < 0.05, corrected).

**Conclusion:**

Progressive deterioration of GM morphology and SCN exists in HBV-RC patients during advanced disease, displaying thalamus-related causal effects. These findings indicate that bilateral thalamus morphology as well as the thalamus-related network may serve as an *in vivo* biomarker for monitoring the progression of the disease in HBV-RC patients.

## Introduction

Progressive deterioration of neurocognitive function in patients with hepatitis B virus (HBV)-related cirrhosis (HBV-RC) as the disease advances is well-documented (1). However, the exact neurobiological mechanisms, especially disease stage-specific brain alterations in HBV-RC patients, are still not fully understood. Identification of the disease stage-specific brain patterns in cirrhotic patients from the no minimal hepatic encephalopathy (NMHE) stage to the minimal HE (MHE) stage may help to further elucidate the neural mechanisms of MHE occurrence and progression.

Gray matter (GM) morphological disruption is among the most replicated findings in cirrhotic patients with HE, which mainly involves the brain regions of the cortex-basal ganglia-thalamus (CxBGTh) circuit (2, 3). As the cardinal region of the CxBGTh circuit, the thalamus has been reported to have a close relationship with HE progression in patients with HBV-RC by both structure magnetic resonance imaging (sMRI) (3) and a pathological study (4). However, the sequence alteration within the CxBGTh circuit from the NMHE to the MHE stage and the potential causal relationship of the thalamus and other brain regions of the CxBGTh circuit are still open questions.

Recently, structural covariance network (SCN) analysis has been developed to identify the unique synchronization patterns among different regions using cross-sectional T1-weighted imaging data (5). Meanwhile, causal network of structural covariance (CaSCN) analysis sequences the structure images in a pseudo-time series according to the disease progression and can map the causal relationships among regions using signed-path coefficient Granger causality (GC) analysis (6). Both methods have been successfully used to investigate the structural network as well as the causal relationship among SCN components in patients with major depressive disorder (7), generalized anxiety disorder (8, 9), and schizophrenia (10, 11). However, to date, no study has used SCN and CaSCN analysis on cirrhotic patients; thus, the SCN and the potential causal relationships among the aberrant SCN components in cirrhotic patients with NMHE and MHE are still unclear.

To fill the gaps, the present study aimed (1) to investigate the disease stage-specific GM morphology alterations in cirrhotic patients with NMHE and MHE and to map the potential relationship between the aberrant GM morphology and the psychometric hepatic encephalopathy score (PHES); (2) to explore the potential causal relationships among the regions with aberrant GM morphology; and (3) to investigate the disease stage-specific SCN alterations in cirrhotic patients with NMHE and MHE. Based on previous studies (2, 12, 13), we put forward several hypotheses: (1) as the cardinal part of the CxBGTh circuit, the thalamus would be the initially involved region in cirrhotic patients, which would correlate with the PHES; (2) as the disease advanced, the abnormal brain regions would expand from the thalamus to other parts of the CxBGTh circuit, and the thalamus would have a causal effect on other brain regions; and (3) as the disease progressed, the thalamus-associated SCN would gradually be destroyed.

## Materials and Methods

### Subjects

The study utilized the Strengthening the Reporting of Observational Studies in Epidemiology (STROBE) cross sectional reporting guidelines (14) and approved by the local Research Ethics Committee. A total of 30 HBV-RC patients, including 17 HBV-RC patients with no MHE (NMHE). Details of demographic, clinical data and exclusion criteria about participants were provided in Supplementary Material.

### Neuropsychological Exams

The severity of neurocognitive dysfunction was assessed by PHES same as our previous papers (15, 16). More details were provided in Supplementary Material.

### MRI Data Acquisition

The fast field echo (FFE) three-dimensional T1-weighted (3D-T1WI) data were acquired by a 1.5-T MR scanner (Achieva Nova Dual; Philips Medical Systems, Best, Netherlands) with a 16-channel neurovascular coil. Additionally, we acquired other sequences to exclude other intracranial diseases. Detailed imaging parameters were provided in Supplementary Material.

### MRI Data Preprocessing

**Figure 1:**
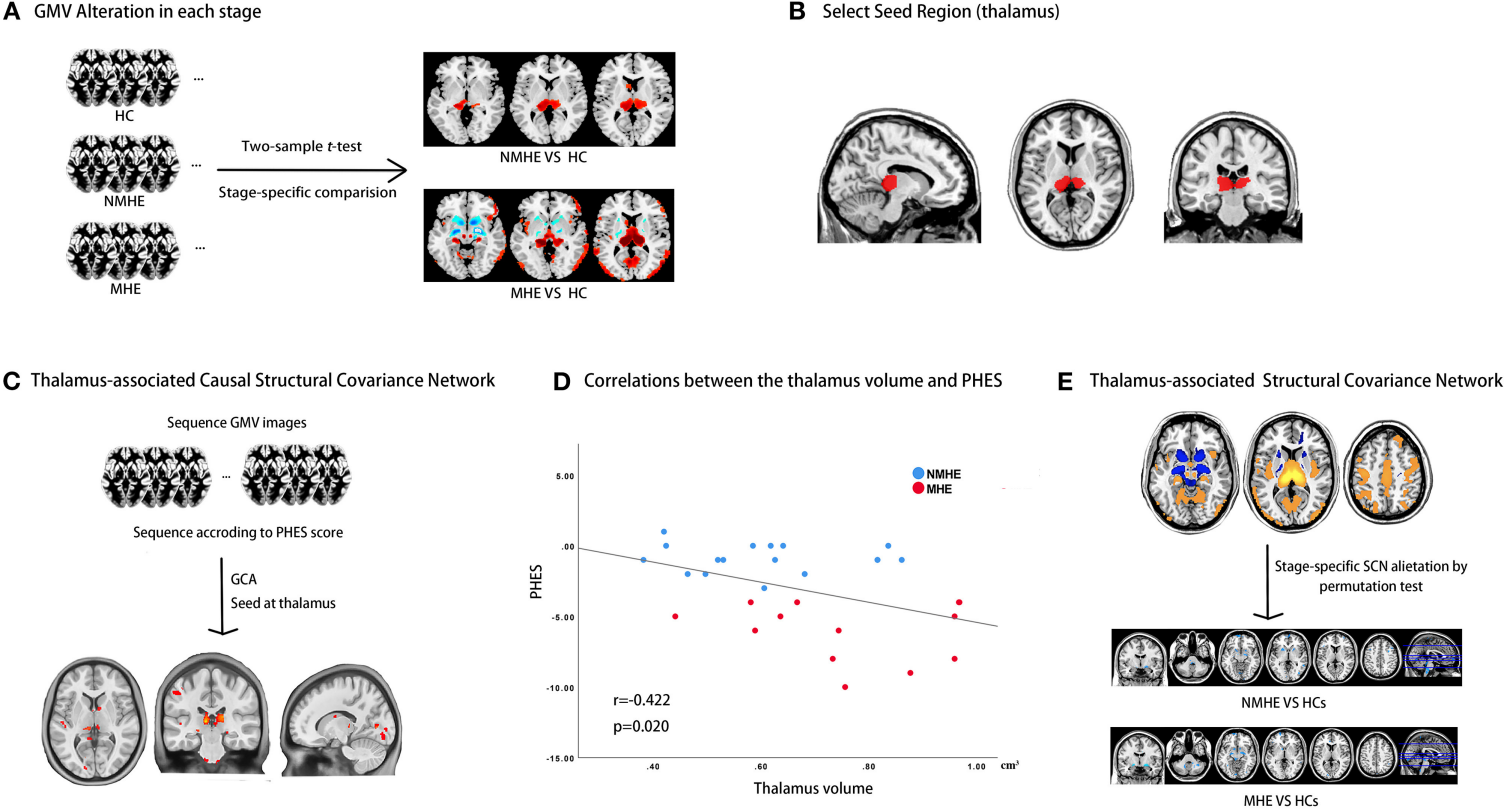
Study design schematic. **(A)** HBV-RC patients were categorized into NMHE and MHE according to PHES; overall and stage-specific gray matter (GM) alterations were calculated by comparing overall cirrhotic patients, NMHE patients, and MHE patients with HCs separately. **(B)** The bilateral thalamus was extracted as the seed region based on prior analysis for subsequent causal network of structural covariance (CaSCN) analysis and structural covariance network (SCN) analysis. **(C)** The thalamus-associated CaSCN was constructed, and sequenced alterations related to the thalamus were derived during the progression of MHE. **(D)** The correlation between the thalamus volume and PHES in HBV-RC patients was evaluated by Spearman's correlation analysis. **(E)** The thalamus-associated SCN was constructed, and stage-specific SCN alterations were calculated by a permutation test. GMV, gray matter volume; HC, healthy control; NMHE, patients without MHE; MHE, minimal hepatic encephalopathy; PHES, psychometric hepatic encephalopathy score; GCA, granger causality analysis; SCN, structural covariance network.

We preprocessed the original 3D-T1WI data using the Computational Anatomy Toolbox (CAT12; http://www.neuro.uni-jena.de/cat/). Details about preprocessing 3D-T1WI data were provided in Supplementary Material. The total intracranial volume (TIV) for each participant was derived in this step. Figure 1 shows the schematic summary of the analytical steps.

### Voxel-Based Morphometry (VBM) Analysis

#### Overall GM Alteration and Disease Stage-Specific GM Alteration

A two-sample *t*-test was performed in SPM12 to estimate the overall GM differences between HBV-RC patients and HCs. Subsequently, each subgroup (NMHE and MHE) was compared with HCs using the same strategy to estimate the stage-specific GM alterations. We included TIV, age, sex, and formal education years as covariates to remove their effects at each comparison. The threshold in the comparison between patients with HBV-RC and HCs was set as, false discovery rate (FDR) < 0.01. To obtain more possible alternative voxels in a specific stage, the threshold for comparison between each subgroup of patients and HCs was set as FDR < 0.05.

#### Thalamus-Based CaSCN in Patients With HBV-RC

Based on the initial morphological changes detected in the bilateral thalamus in the NMHE stage of HBV-RC, thalamus-based CaSCN analysis was performed to investigate the probable sequential patterns of thalamus swell and other GM region alterations in patients with HBV-RC following a previous study (6). First, all GM images of HBV-RC patients were sequenced by their PHES as pseudo-time series. Then, we selected the thalamus as the seed region based on the two-sample *t*-test comparison between NMHE and HCs (FDR < 0.05). Subsequently, voxel-wise CaSCN analyses with the thalamus serving as the seed region were conducted using the signed path coefficient Granger causality analysis (GCA) implemented in the REST 1.8 package (REST; http://www.restfmri.net/forum/REST_V1.8). We only adopted the GC values of X to Y in this research due to the initial alteration of the thalamus detected in the NMHE stage. The original voxel-wise GC map was transformed as a *Z* score map. We conducted a two-sample *t*-test with a primary threshold (*t* > 1.96, *p* < 0.05), and then, the permutation tests (1,000 iterations) were used to correct the result (threshold α = 0.05).

#### Correlations Between the Bilateral Thalamus Volume and PHES in HBV-RC Patients

Spearman's correlation analysis was performed to measure the relationship between the thalamic volume and PHES performance in all patients. The threshold was set as *p* < 0.05, and this analysis was conducted in IBM SPSS v. 25.0.

#### Thalamus-Related SCN Alteration in HBV-RC Patients

We also performed thalamus-based SCN analysis to detect disease stage-specific thalamus-related structural network changes in HBV-RC patients following a previous procedure (17). First, we extracted the GM values in the seed region (bilateral thalamus) from NMHE patients, MHE patients, and HCs, respectively. The extracted GM values were used to construct the VBM-SCN *t* maps (FDR < 0.01). Subsequently, a linear interaction analysis was performed to measure the SCN alteration in pairwise comparison (6) (overall SCN alterations between HBV-RC patients and HCs, as well as comparison of each subgroup [NMHE and MHE] with HCs to estimate the stage-specific SCN alterations), and permutation tests (1,000 iterations) (18) were used to correct the results (threshold α = 0.05). In this step, we also measured TIV, age, sex, and education level as covariates to remove their effects.

## Results

### Demographics and Clinical Characteristics

There were no significant differences among the three groups in terms of age, sex, or formal years of education (*p* = 0.479, *p* = 1, *p* = 0.054, respectively). There were no significant differences between NMHE and MHE in Child–Pugh stage (*p* = 0.265), total serum bilirubin (NMHE/MHE, 38.11 ± 12.16/47.00 ± 11.82, unit: mg/dl, *p* = 0.198), prothrombin time (NMHE/MHE, 15.92 ± 1.96/16.46 ± 1.95, unit: seconds, *p* = 0.363), alanine aminotransferase (NMHE/MHE, 57.65 ± 55.25/49.615 ± 34.19, unit: IU/L, *p* = 0.837), and aspartate aminotransferase (NMHE/MHE, 49.12 ± 24.92/71.54 ± 51.15, unit: IU/L, *p* = 0.263); however, MHE patients (32.24 ± 6.29 mg/dl) had lower serum albumin than NMHE patients (37.04 ± 6.16 mg/dl, *p* < 0.05). Both patient subgroups had poorer PHES performances than HCs, while NMHE patients had better PHES performances than MHE patients (*p* < 0.05) (Table 1). There was no difference in TIV among the three groups (*p* = 0.486) (Table 1).

**Table 1:** Demographic and neuropsychological characteristics of HCs and cirrhotic patients.

	**HCs**	**NHME**	**MHE**	***p***
Age (year)	44.763 ± 9.6628	43.118 ± 8.9434	47.462 ± 11.0876	0.479
Sex (male/female)	31/7	14/3	11/2	1
Education level (year)	11.42 ± 3.019	10.71 ± 3.216	9.08 ± 3.883	0.054
Child–Pugh stage (A/B/C)	NA	11/5/1	5/5/3	0.265
Albumin (mg/dl)	NA	37.0353 ± 6.15903	32.2385 ± 6.28882	0.045
Total bilirubin (mg/dl)	NA	38.11 ± 12.157	47.00 ± 11.817	0.198
Prothrombin time (s)	NA	15.918 ± 1.963	16.462 ± 1.954	0.363
PHES (−15–5)	0.11 ± 0.981 (−2–1)	−0.88 ± 0.993 (−3–1)	−6 ± 2.028 (−10 to −4)	<0.05*^, †^,#
ALT (IU/L)	NA	57.65 ± 55.25	49.615 ± 34.19	0.837
AST (IU/L)	NA	49.12 ± 24.92	71.54 ± 51.15	0.263
TIV (cm^3^)	1,435.990 ± 20.408	1,472.680 ± 33.026	1,476.565 ± 37.840	0.486
Thalamus volume (cm^3^)	0.443 ± 0.073	0.607 ± 0.154	0.760 ± 0.175	<0.05^*, †, #^

*HCs, healthy controls; NMHE, patients without MHE; MHE, minimal hepatic encephalopathy; PHES, psychometric hepatic encephalopathy score; ALT, alanine aminotransferase; AST, aspartate aminotransferase; TIV, total intracranial volume*.

The markers ^*^, †, and # indicate significant differences in neurological performance between MHE and HCs, NMHE and HCs, and MHE and NMHE, respectively.

### Disease Stage-Specific GM Alteration Pattern in HBV-RC Patients

**Figure 2:**
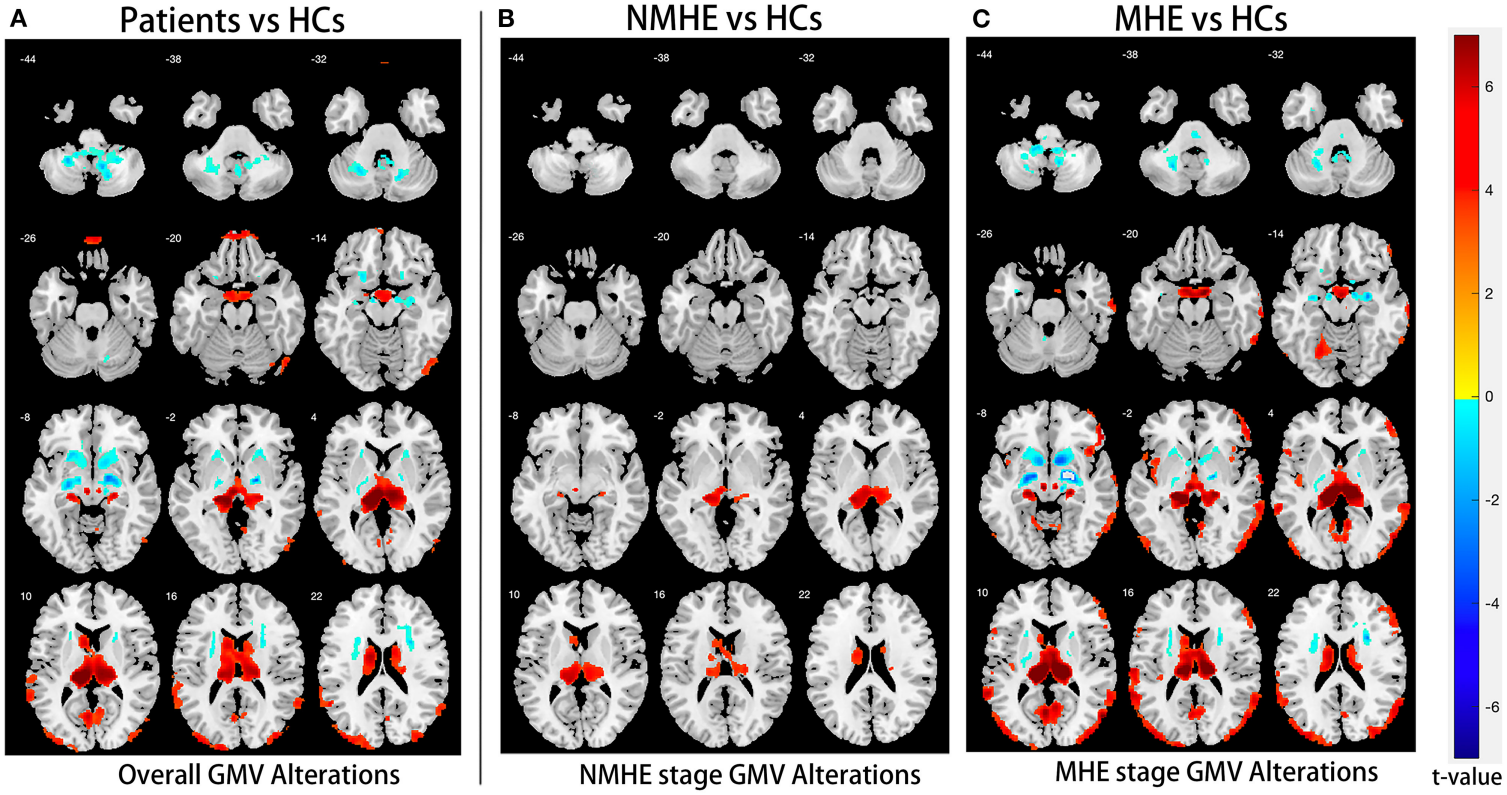
Overall and stage-specific gray matter alterations. Overall GM alterations in all cirrhotic patients mainly involved the cortex-basal ganglia-thalamus (CxBGTh) circuit and the thalamus–cerebellum circuit compared to HCs (**A**, false discovery rate [FDR] < 0.01). Meanwhile, stage-specific GM alteration started from the bilateral thalamus and the left caudate in the NMHE stage (compared with HCs, FDR < 0.05) **(B)**, extending to other regions of the CxBGTh circuit and the thalamic-cerebellum circuit (including the bilateral basal ganglia, the bilateral cerebellum, and the cortex) in the MHE stage (compared with HCs, FDR < 0.05) **(C)**. The *t*-values were represented by the colors shown in the color bar. GM, gray matter; HCs, healthy controls; NMHE, patients without MHE; MHE, minimal hepatic encephalopathy.

**Figure 3:**
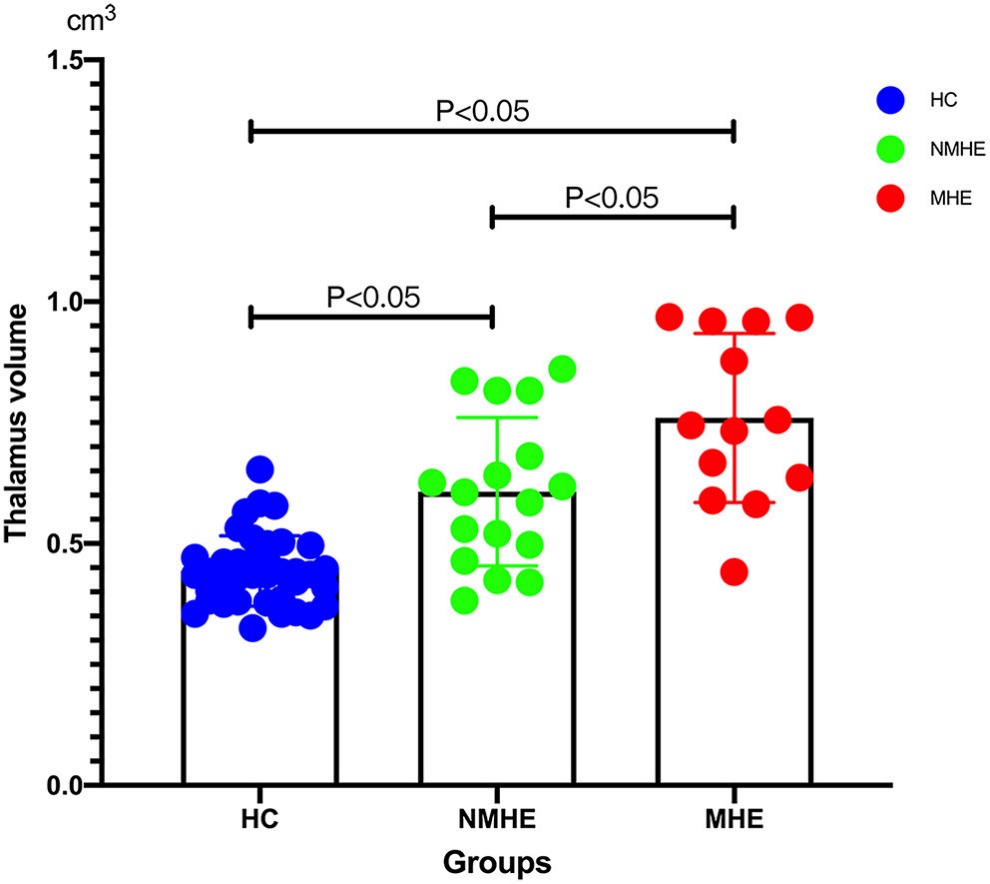
Thalamus volume differences among three groups. Thalamic volume displayed progressive swelling as the disease advanced in cirrhotic patients compared with HCs. HCs, healthy controls; NMHE, patients without MHE; MHE, minimal hepatic encephalopathy.

Compared to HCs, NMHE patients had significantly increased volume in the bilateral thalamus extending to the left caudate nucleus (FDR < 0.05), but other brain regions remained stable. In the MHE stage, the thalamus continued to swell (compared to NMHE, *p* < 0.05, Bonferroni corrected); in addition, the abnormal regions extended to the bilateral basal ganglia, the bilateral cerebellum, and the cortex (compared to HCs, FDR < 0.05). Specifically, the basal ganglia (bilateral lentiform nucleus, bilateral putamen, right amygdala, and right caudate nucleus) and cerebellum appeared to atrophy, while the cortex (including the bilateral temporal lobe, bilateral occipital lobe, bilateral superior frontal lobe, and left parietal lobe) appeared swollen compared to that of HCs (FDR < 0.05). The results are shown in Figures 2, 3, and details are provided in Supplementary Tables 1, 2.

### Probable Sequential Patterns of Thalamus Swelling and Other GM Region Alterations in HBV-RC Patients

**Figure 4:**
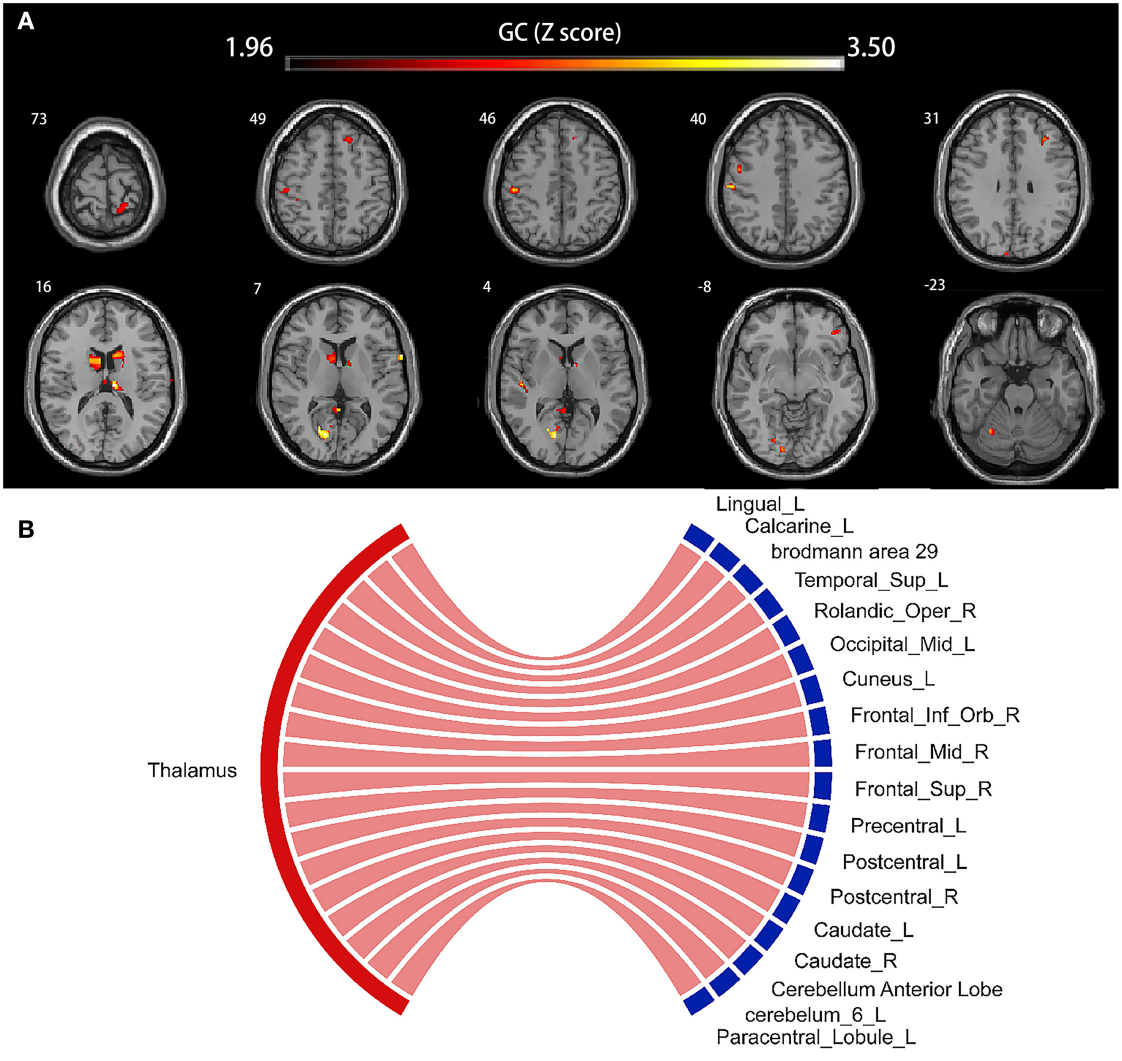
Voxel-based causal network of structural covariance (CaSCN). Thalamus swelling had causal effects on the left lingual gyrus, the left calcarine sulcus, Brodmann area 29, left middle occipital gyrus, left cuneus, left superior temporal gyrus, right Rolandic operculum, right frontal inferior operculum, right frontal middle gyrus, right frontal superior gyrus, left precentral gyrus, left paracentral lobule, bilateral postcentral gyrus, bilateral caudate nucleus, bilateral thalamus, and cerebellum (*p* < 0.05, corrected). Significant regions were overlaid onto the Montreal Neurological Institute (MNI) template, and the Granger causality value is represented by the colors shown in the color bar **(A)**. The Sankey and Chord were also used to display the causal effects intuitively **(B)**; the flow from the bilateral thalamus (red label) to the thalamus-affected regions (blue label) is colored in red according to the positive causal effects. GC, Granger causality; L, left; R, right; Sup, supper; Oper, opercularis; Mid, middle; Inf, inferior; Orb, orbitalis.

The voxel-wise GC map showed a positive GC value from the thalamus to the occipital lobe (Lingual_L, GC value = 2.91; Calcarine_L, GC value = 3.62; Brodmann area 29, GC value = 2.99; Occipital_Mid_L, GC value = 2.68; Cuneus_L, GC value = 3.11), Temporal_Sup_L (GC value = 3.13), the frontal lobe (Rolandic_Oper_R, GC value = 3.34; Frontal_Inf_Orb_R, GC value = 2.57; Frontal_Mid_R, GC value = 2.86; Frontal_Sup_R, GC value = 2.67; Precentral_L, GC value = 2.57; Paracentral_Lobule_L, GC value = 2.28), bilateral postcentral gyrus (Postcentral_L, GC value = 2.63; Postcentral_R, GC value = 2.34), bilateral caudate nucleus (Caudate_L, GC value = 2.87; Caudate_R, GC value = 3.52), bilateral thalamus (Thalamus_L, GC value = 3.62; Thalamus_R, GC value = 2.59), and cerebellum (Cerebellum Anterior Lobe, GC value = 2.61; Cerebelum_6_L, GC value = 2.67) (all *p* < 0.05, corrected). The results are shown in Figure 4, and details are provided in Supplementary Table 3.

### Thalamus Volume and PHES Performance in HBV-RC Patients

**Figure 5:**
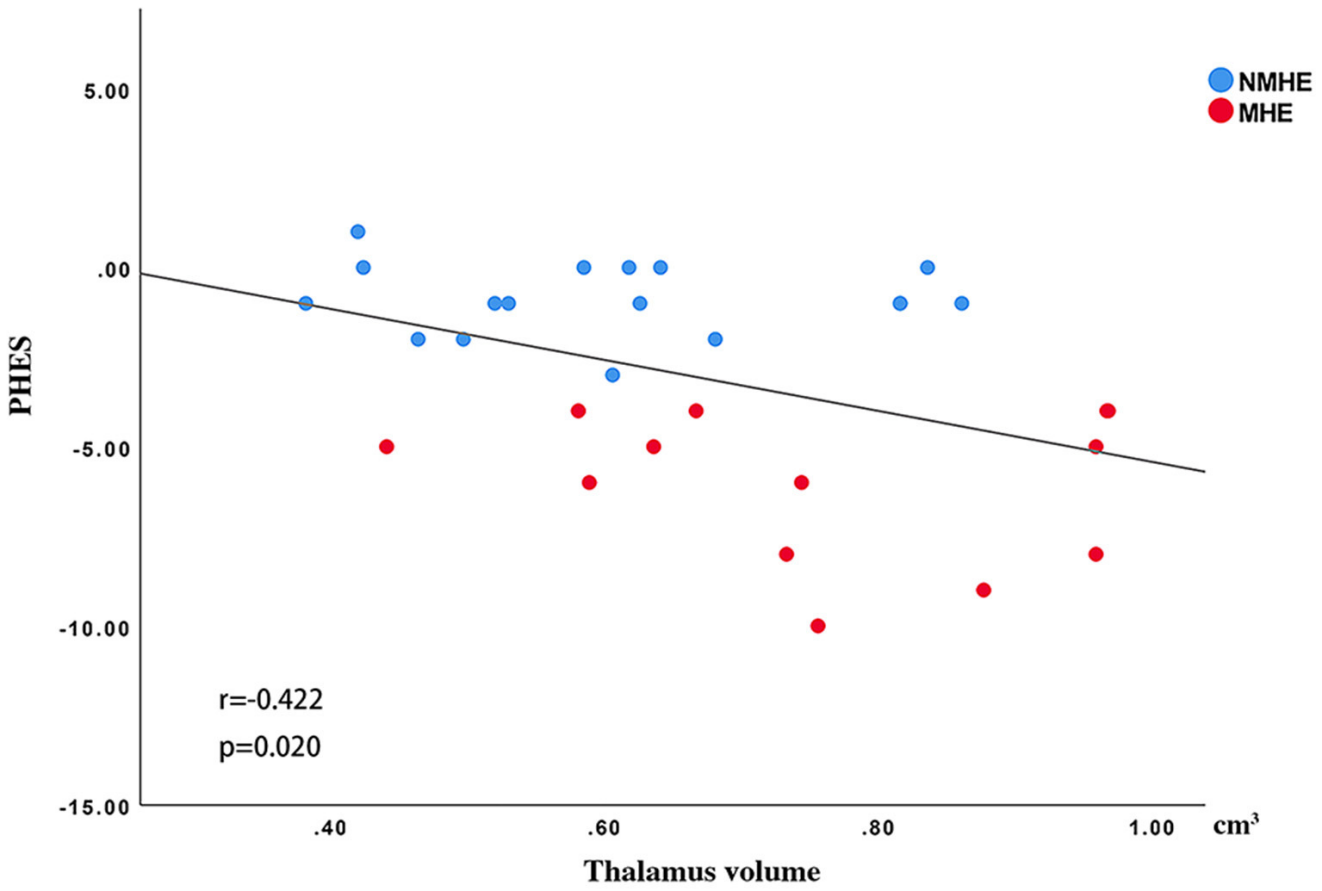
Spearman's correlations between the thalamus volume and PHES. The thalamus volume was negatively correlated with PHES performance in all cirrhosis patients. NMHE, patients without MHE; MHE, minimal hepatic encephalopathy; PHES, psychometric hepatic encephalopathy score.

There was a significantly negative Spearman's correlation between the thalamus volume and PHES (*r* = −0.422, *p* < 0.05) in all HBV-RC patients (Figure 5).

### Disease Stage-Specific Thalamus-Associated SCN Alterations in HBV-RC Patients

**Figure 6:**
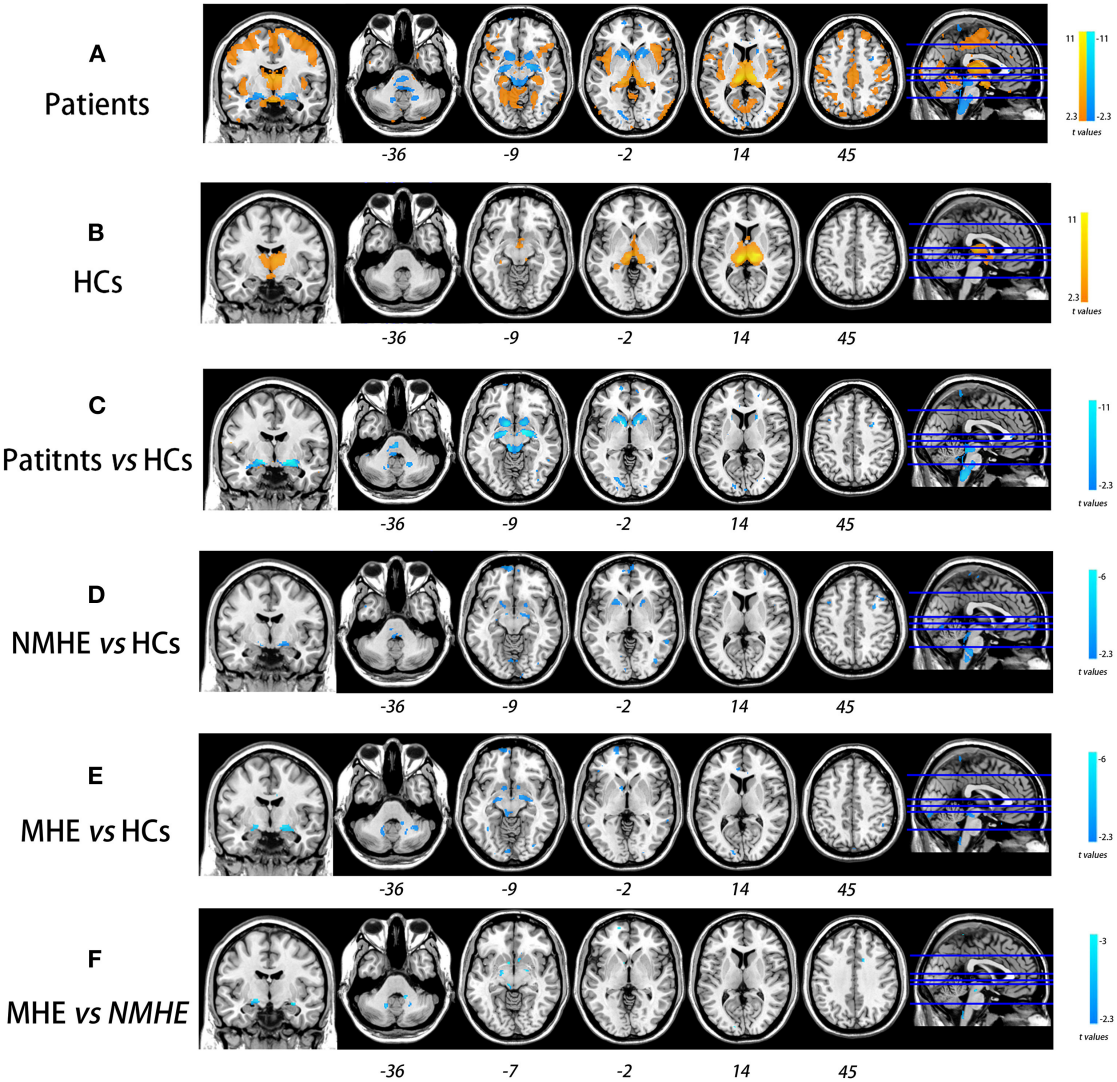
Progressive deterioration of the thalamus-associated structural covariance network (SCN) in HBV-RC patients. Both HC and HBV-RC patients exhibited positive synchronous SCN between the thalamus and cortex **(A,B)**. In HBV-RC patients, negative synchronous SCN between the thalamus and basal ganglia was also observed **(B)**. The HBV-RC patients exhibited enhanced negative synchronous SCN between the thalamus and the bilateral basal ganglia as well as the cortex compared with HCs **(C)**. Furthermore, progressive disruption of thalamus-related SCN was observed in HBV-RC patients as the disease advanced. Specifically, in the NMHE stage, patients showed increased negative synchronization between the basal ganglia and cortex compared to HCs (*p* < 0.01, corrected, **D**), and in the MHE stage, the negative synchronization between the thalamus and basal ganglia increased further compared to HCs **(E)** and NMHE patients **(F)**. The color bar represents the *t*-values. HCs, healthy controls; NMHE, patients without MHE; MHE, minimal hepatic encephalopathy.

In both the HC and HBV-RC patient groups, a positive synchronous structural covariance network was observed between the thalamus and cortex (Figures 6A,B); however, a negative synchronous structural covariance network between the thalamus and basal ganglia was also observed in HBV-RC patients but not in the HC group (Figures 6A–C). Furthermore, progressive disruption of thalamus-related SCN was observed in HBV-RC patients as the disease advanced. Specifically, in the NMHE stage, patients showed increased negative synchronization between the basal ganglia and cortex compared to HCs (*p* < 0.01, corrected), and in the MHE stage, the negative synchronization between the thalamus and basal ganglia was increased further compared to HCs and NMHE patients (all *p* < 0.01, corrected, Figures 6D–F). The results are shown in Figure 6, and details are provided in Supplementary Table 4.

## Discussion

Patients with HBV-RC underwent gradual damage in GM morphology as the disease advanced, which began at the bilateral thalamus in the NMHE stage and extended to the bilateral basal ganglia, cerebellum, and cortex in the MHE stage. Furthermore, the thalamic abnormalities had causal effects on other parts of the CxBGTh circuit and the thalamus–cerebellum circuit in patients with HBV-RC. Interestingly, thalamic abnormalities were correlated with poor PHES performance in all HBV-RC patients. Moreover, progressive disruption of thalamus-related SCN was also observed in HBV-RC patients along with disease advancement. Specifically, in the NMHE stage, patients displayed increased negative synchronization between the bilateral thalamus and bilateral basal ganglia, cerebellum, and cortex compared to HCs, while in the MHE stage, such negative synchronization was enhanced further compared with HCs and NMHE patients. These findings indicate that bilateral thalamus morphology as well as thalamus-related networks may serve as an *in vivo* biomarker for monitoring the progression of the disease in patients with HBV-RC.

### Stage-Specific Alterations in GM Morphology in Patients With HBV-RC

The disease stage-specific GM alterations observed in this study were consistent with previous cross-sectional studies (2, 19). By comparing the structural magnetic resonance imaging of 24 cirrhotic patients without MHE, 23 cirrhotic patients with MHE, 24 cirrhotic patients with overt HE, and 33 healthy controls, Tao et al. (2) revealed that the thalamic volume increased in a stepwise manner in patients with advanced stages of hepatic encephalopathy compared to healthy subjects. Our present results were consistent with previous findings on the stage-specific thalamic volume alterations. Moreover, our study revealed that the aberrant regions expanded from the thalamus to other regions of the CxBGTh circuit (including the basal ganglia, the cortex, and the cerebellum) as the disease advanced in HBV-RC patients (in the MHE stage). Such stage-specific brain changes in cirrhotic patients were also reported using other imaging modalities. In an arterial spin labeling study by Zheng et al. (20), abnormal blood flow was initially found in the bilateral thalamus (in the non-HE stage), which then extended to the insula, cingulate cortex, occipital lobe, parietal lobe, temporal lobe, and putamen as the disease advanced (at the MHE stage). Using positron emission tomography (PET), Iversen et al. (21) also observed higher cerebral blood flow (CBF) in the thalamus in cirrhotic patients without HE, while in the HE stage, the CBF in the thalamus further increased, and the abnormal regions expanded to the parietal lobe and occipital lobe. Taken together, these findings suggest that the thalamus may be the initially involved region during the progression of HBV-RC. This finding is not surprising, given that the liver is a major site for thiamine synthesis and storage in human (12, 22), thereby chronic liver failure or injury caused by HBV-RC would result in thiamine deficiency (12, 23). Previous studies had demonstrated that the thalamus is among the most sensitive brain regions to thiamine deficiency (24, 25).Thus, thiamine deficiency could cause neuron damage and microglial–neuroimmune activation in the thalamus, but not other brain regions (24, 26), which manifests as swelling during the initial stage of the disease.

### The Causal Effect of the Thalamus on Cortex and Cerebellum Changes

Furthermore, we found that the increase of the thalamus volume had a causal effect on the swelling of the cortex and cerebellum as well as basal ganglia atrophy. In other words, the swollen thalamus contributed to neural damage in the cortex, cerebellum, and basal ganglia. The thalamus plays important roles in the CxBGTh circuit (13) and thalamic–cerebellar circuit (27). Damage to the thalamus could cause disorder in the nucleus accumbens (NAcc)–ventral pallidum–mediodorsal thalamus–cortex pathway (27). To compensate for that, an alternative pathway (NAcc–substantia nigra pars reticulata [SNr]–ventromedial thalamus–cortex) is activated (28). We speculate that the alternative pathway would restrain the function of the ganglia and cause disuse atrophy of the ganglia (29). The accumulation of neurotransmitters causes persistent activation of the cortex, which leads to cortex swelling sequentially. The thalamus and the cerebellum contact not only by intensive disynaptic projection (30) but also by the glutamate–nitric oxide (NO)–cGMP pathway (27). The dysfunction of the thalamus leads to the increased expression of cFOS gene in the cerebellum, and then, the overexpressed cFOS gene reduces the Purkinje cells in the cerebellum (31, 32), which in turn leads to cerebellum atrophy. Further studies are needed to validate this hypothesis in future.

### Thalamic Volume Was Correlated With PHES in Patients With HBV-RC

Interestingly, we observed that increased thalamic volume had a negative correlation with PHES in patients with HBV-RC. As far as we know, the thalamus is a transfer and integration station in the central nervous system (33). Abnormalities in the thalamus can result in high-level cognitive dysfunctions including deterioration of memory, execution dysfunction, apatheia, and dyskinesia (34), which in turn lead to the poor PHES performance of HBV-RC patients. This finding suggests that the alteration of thalamus volume may be a sensitive index to monitor the disease progression.

### Disease Stage-Specific SCN Alteration Pattern in Patients With HBV-RC

In addition to the progressive impairment of GM morphology, we also observed disease stage-specific SCN alteration in patients with HBV-RC, which mainly involved the CxBGTh circuit and the thalamus–cerebellum circuit. SCN damage in MHE has been reported previously by Zou et al. (35), who found that MHE patients had abnormal small-world properties of the brain SCN, consistent with our findings. Furthermore, the present study complemented prior knowledge that SCN impairment happens earlier in patients with HBV-RC (in the NMHE stage). We found that the enhanced negative connectivity within the CxBGTh circuit and the thalamus–cerebellum circuit had already appeared in the NMHE stage; such abnormalities progressed in severity and extent in the MHE stage. These findings indicated the CxBGTh circuit and the thalamus–cerebellum circuit underwent progressive disruption as the disease advanced in patients with HBV-RC, which could serve as a sensitive biomarker to monitor the progression of HBV-RC *in vivo*.

There are several limitations to this study. First, our sample size was small. Though we met the least sample size (*n* > 10) according to the sample size calculation following previous study (36), large-scale research is needed to verify these preliminary findings. Second, the cross-sectional nature of the present study with pseudo-time series posed limitations on inferences of causality. Longitudinal studies would enable us to ascertain a more complete trajectory of GM morphological as well as GM network alterations in HBV-RC patients. Third, body mass index (BMI) was not available in present study. Although BMI preferred to influence the volume of hippocampal (37) and medial orbitofrontal (38), future studies still need to demonstrate their potential influence on the thalamus in patients with HBV-RC. Finally, this preliminary research explored the disease stage-specific GM morphological and network alterations in patients with HBV-RC, and we only included cirrhotic patients with MHE or NMHE; thus, the alterations from MHE to overt HE (OHE) are still an open question. In future, we will recruit cirrhotic patients with OHE to complement such alterations to the OHE stage.

## Conclusion

Our study explored the disease stage-specific GM alteration pattern in HBV-RC patients. Our results provided evidence of progressive disruption in GM morphology in HBV-RC patients as the disease advanced, which started with the bilateral thalamus swelling in the NMHE stage and then extended to the ganglia, cerebellum, and cortex in the MHE stage. Furthermore, the thalamus swelling had a causal effect on the morphological alteration of other brain regions involved in the basal CxBGTh circuit and the thalamus–cerebellum circuit, which also underlay the poor PHES performance in HBV-RC patients. Moreover, the thalamus-related structural network also underwent progressive disruption with disease advancement in HBV-RC patients. Thus, the thalamus may be the hinge of structural alterations and may serve as an important tool for monitoring the progression of HBV-RC.

## Data Availability Statement

The raw data supporting the conclusions of this article will be made available by the authors, without undue reservation.

## Ethics Statement

The studies involving human participants were reviewed and approved by Research Ethics Review Board of Nanfang Hospital, Southern Medical University. The patients/participants provided their written informed consent to participate in this study.

## Author Contributions

SL, ZG, SC, XL, and YQ: drafting, revision of the manuscript for content, including medical writing for content, study concept or design, and analysis or interpretation of data. MY: major role in the acquisition of data. All authors contributed to the article and approved the submitted version.

## Funding

This study has received funding by Natural Scientific Foundation of China (Grant Nos: 81560283 and 81201084) and Natural Science Foundation of Guangdong (Grant No: 2020A1515011332).

## Conflict of Interest

The authors declare that the research was conducted in the absence of any commercial or financial relationships that could be construed as a potential conflict of interest.

## Publisher's Note

All claims expressed in this article are solely those of the authors and do not necessarily represent those of their affiliated organizations, or those of the publisher, the editors and the reviewers. Any product that may be evaluated in this article, or claim that may be made by its manufacturer, is not guaranteed or endorsed by the publisher.
