# Supplementary material for: Progressive Brain Structural Impairment Assessed via Network and Causal Analysis in Patients With Hepatitis B Virus-Related Cirrhosis
Source: Front Neurol. 2022 May 6;13:849571. doi: 10.3389/fneur.2022.849571 (PMC9120530; doi:10.3389/fneur.2022.849571)
Supplement: Supplementary file 1 [file Data_Sheet_1.docx]

**Materials and methods**

***Subjects***

The study utilized the Strengthening the Reporting of Observational Studies in Epidemiology (STROBE) cross sectional reporting guidelines.[1] A total of 30 HBV-RC patients, including 17 HBV-RC patients with no MHE (NMHE) (14 male, 43.12 ± 8.94 years) and 13 MHE patients (11 male, 47.46 ± 11.09 years), were recruited for this study. Thirty-eight sex-, age-, and education-matched healthy controls (HCs) (31 male, 44.76 ± 9.66 years) were also recruited for this study. All patients needed to have one or more types of evidence to diagnose HBV-RC, such as biochemical or imaging findings, biopsy, or clinical examination. The exclusion criteria were as follows: MRI contraindications, other types of hepatitis cirrhosis, severe cranial injury, an alcohol or drug abuse history, neuropathy or psychosis history, visual impairment, or being left-handed. The Child–Pugh score was used to determine the severity of the liver statuses of HBV-RC patients (Child–Pugh stage A/B/C; NMHE, 11/5/1; MHE, 5/5/3). Albumin, total bilirubin, prothrombin time, alanine aminotransferase and aspartate aminotransferase were also recorded for all HBV-RC patients. This prospective study was approved by the local Research Ethics Committee, and each participant signed an informed consent form before the study.

***Neuropsychological exams***

The severity of neurocognitive dysfunction was assessed by PHES as detailed in our previous papers.[2, 3] All participants completed five subtests of the PHES exam: number connection tests A and B (NCT-A, NCT-B; both were measured in seconds), the serial dotting test (SDT, measured in seconds), the digit symbol test (DST, measured in numbers), and the line tracing test (LTT, measured in seconds as well as the accuracy rate). In the NCT-B, we replaced all English characters with Chinese characters as some of the participants could not understand the English alphabet.[2] The details of PHES calculation can be found in our previous reports.[2, 3] Finally, the patients with a score < 4 were included in the MHE group, while those with a score > 4 were included in the NMHE group.

***MRI data acquisition***

The fast field echo (FFE) three-dimensional T1-weighted (3D-T1WI) data were acquired by a 1.5-T MR scanner (Achieva Nova Dual; Philips Medical Systems, Best, Netherlands) with a 16-channel neurovascular coil. Detailed imaging parameters were as follows: repletion time (TR) = 25 ms, echo time (TE) = 4.1 ms, flip angle = 30°, FOV = 230 mm^2^, matrix size = 231 × 232, section thickness = 1 mm without gap. Additionally, we acquired other sequences (e.g., two-dimensional T1-weighted [2D-T1WI]: TR = 600 ms, TE = 29 ms; T2 fluid-attenuated inversion recovery (FLAIR) images: TR = 6000 ms, TE = 120 ms, inversion time = 2000 ms) to exclude other intracranial diseases.

***MRI data preprocessing***

We preprocessed the original 3D-T1WI data using the Computational Anatomy Toolbox (CAT12; <http://www.neuro.uni-jena.de/cat/>), which was implemented in statistical parametric mapping (SPM12; <https://www.fil.ion.ucl.ac.uk/spm/software/spm12/>). First, we checked the quality of all data and artificially re-oriented their origins to the anterior commissure. Second, we normalized the data into Montreal Neurological Institute (MNI) space using the diffeomorphic anatomical registration through the exponentiated lie algebra (DARTEL) method.[4] Thereafter, all data were segmented into GM, white matter, and cerebrospinal fluid based on the tissue probability maps; the segmented data were then resampled to a cubic voxel size of 1.5 mm^3^. The total intracranial volume (TIV) for each participant was derived in this step. Finally, we used a 6-mm full width at half maximum (FWHM) isotropic Gaussian kernel to smooth all segmented GM images for the subsequent analysis.

Table S1. GM alterations in Patients relative to HCs.

| Brain regions | MNI coordinates (x, y, z) | | | Peak t-value | Number of voxels |
| --- | --- | --- | --- | --- | --- |
| Increased GM |  |  |  |  |  |
| Thalamus | -18 | -28 | 8 | 7.8 | 3543 |
| Temporal_Inf_R | 66 | -14 | -27 | 5.87 | 184 |
| Postcentral_L | -38 | -15 | 50 | 5.64 | 220 |
| Calcarine_L | -4 | -68 | 12 | 5.6 | 298 |
| Paracentral_Lobule_L | -3 | -22 | 52 | 4.96 | 207 |
| Superior Frontal Gyrus | -8 | 62 | -22 | 5.39 | 218 |
| Occipital_Mid_L | -16 | -100 | 18 | 4.54 | 304 |
| Precentral_R | 54 | 8 | 45 | 4.88 | 209 |
| Occipital_Mid_R | 39 | -87 | 18 | 4.81 | 395 |
| Occipital_Inf_R | 50 | -70 | -18 | 4.61 | 111 |
| Temporal_Sup_L | -62 | -30 | 14 | 4.50 | 239 |
| Superior Temporal Gyrus_R | 62 | -54 | 18 | 4.4 | 204 |
| Decreased GM |  |  |  |  |  |
| Lentiform Nucleus_R | 20 | -8 | -3 | -6.93 | 189 |
| Lentiform Nucleus_L | -26 | -14 | -9 | -5.52 | 145 |
| Caudate_R | 14 | 10 | -8 | -4.92 | 53 |
| Putamen_R | 24 | 21 | -3 | -4.68 | 20 |
| Amygdala_R | 33 | 2 | -28 | -5.64 | 28 |
| Putamen_L | -21 | 20 | -8 | -5.13 | 116 |
| Cerebelum_6_L | -24 | -58 | -33 | -5.51 | 103 |
| Cerebelum_8_L | -24 | -50 | -46 | -5.58 | 137 |
| Cerebelum_9_R | 15 | -50 | -54 | -5.53 | 331 |

Abbreviations: HCs, healthy controls; MNI, Montreal Neurological Institute; L, left; R, right; GM, gray matter

Note: the threshold set as p<0.01, false discovery rate corrected

Table S2. GM alterations in each subgroup in patients relative to HCs.

| Brain regions | MNI coordinates (x, y, z) | | | Peak t-value | Number of voxels |
| --- | --- | --- | --- | --- | --- |
| NMHE vs HCs |  |  |  |  |  |
| Thalamus | -19.5 | -28.5 | 1.5 | 6.1914 | 956 |
| Caudate_L | -13.5 | -4.5 | 22.5 | 4.4411 | 251 |
|  |  |  |  |  |  |
| MHE vs HCs |  |  |  |  |  |
| Frontal_Inf_Tri_R | 54 | 40.5 | -6 | 5.5368 | 857 |
| Frontal_Inf_Tri_L | -55.5 | 19.5 | 7.5 | 5.3926 | 718 |
| Frontal_Med_Orb_L | 0 | 60 | -22.5 | 6.1908 | 289 |
| Frontal_Mid_Orb_L | -18 | 30 | -27 | 5.4523 | 118 |
| Frontal_Inf_Orb_L | -52.5 | 37.5 | -6 | 4.3045 | 95 |
| Frontal_Sup_R | 25.5 | 66 | 22.5 | 4.216 | 50 |
| Temporal_Sup_L | -63 | -40.5 | 4.5 | 5.2923 | 551 |
| Temporal_Inf_L | -66 | -21 | -24 | 4.8621 | 269 |
| Temporal_Mid_L | -64.5 | -6 | -16.5 | 4.0389 | 100 |
| Precentral_L | -34.5 | -18 | 49.5 | 4.3196 | 612 |
| Precentral_R | 30 | -22.5 | 54 | 3.6364 | 533 |
| Parietal_Inf_L | -40.5 | -48 | 54 | 5.1576 | 626 |
| Parietal_Sup_L | -21 | -70.5 | 49.5 | 4.528 | 157 |
| Supp_Motor_Area_R | 4.5 | -40.5 | 55.5 | 6.4457 | 459 |
| Supp_Motor_Area_L | -3 | 36 | 28.5 | 3.6282 | 111 |
| Thalamus_R/L | -15 | -27 | 10.5 | 8.9479 | 1846/1718 |
| Precuneus | 57 | -72 | 10.5 | 6.4268 | 2469 |
| Occipital_Mid_L | -33 | -91.5 | 21 | 5.3801 | 1361 |
| Fusiform_R | 21 | -52.5 | -12 | 5.1873 | 211 |
| Heschl_R | 42 | -18 | 9 | 5.0829 | 186 |
| Angular_L | -45 | -58.5 | 27 | 3.8062 | 113 |
| Cerebelum_9_R | -10.5 | -39 | -45 | -6.793 | 1325 |
| Cerebelum_8_R | 18 | -73.5 | -43.5 | -3.5538 | 40 |
| Hippocampus_L | -12 | -7.5 | -10.5 | -7.8404 | 104 |
| ParaHippocampal_R | 25.5 | -18 | -25.5 | -3.5408 | 16 |
| ParaHippocampal_L | -25.5 | -19.5 | -25.5 | -3.6471 | 14 |
| Putamen_R | 15 | -4.5 | -10.5 | -8.2649 | 376 |
| Putamen_L | -9 | 7.5 | -7.5 | -5.1609 | 371 |
| Cingulum_Ant_R | 16.5 | 48 | 7.5 | -3.7112 | 98 |
| Frontal_Sup_L | -13.5 | 19.5 | 52.5 | -4.1651 | 74 |
| Lingual_L | -15 | -82.5 | -1.5 | -4.8018 | 69 |
| Frontal_Sup_Medial_L | -13.5 | 39 | 19.5 | -4.3555 | 37 |
| Amygdala_R | 36 | -4.5 | -24 | -4.0793 | 36 |
| Cuneus_L | -12 | -85.5 | 13.5 | -5.034 | 33 |
| Claustrum | -24 | 12 | 18 | -4.6287 | 32 |
| Calcarine_R | 19.5 | -90 | -1.5 | -3.8427 | 28 |
| Postcentral_L | -52.5 | -10.5 | 19.5 | -3.6799 | 27 |
| Occipital_Mid_L | -27 | -70.5 | 0 | -3.7237 | 14 |

Abbreviations: NMHE, no minimal hepatic encephalopathy; MHE, minimal hepatic encephalopathy; HCs, healthy controls; MNI, Montreal Neurological Institute; L, left; R, right; GM, gray matter

Note: the threshold set as p<0.05, false discovery rate corrected

Table S3. The thalamus-associated CaSCN

| Brain regions | MNI coordinates (x, y, z) | | | GC (Z score) | Number of voxels |
| --- | --- | --- | --- | --- | --- |
| Lingual_L | -15 | -87 | -10.5 | 2.9149 | 92 |
| Calcarine_L | -15 | -73.5 | 6 | 3.6202 | 265 |
| brodmann area 29 | -1.5 | -48 | 7.5 | 2.9943 | 35 |
| Occipital_Mid_L | -28.5 | -85.5 | 18 | 2.6817 | 20 |
| Cuneus_L | -9 | -88.5 | 21 | 3.1172 | 41 |
| Temporal_Sup_L | -49.5 | -18 | 4.5 | 3.1396 | 55 |
| Rolandic_Oper_R | 64.5 | 10.5 | 7.5 | 3.344 | 35 |
| Frontal_Inf_Orb_R | 39 | 39 | -7.5 | 2.57 | 38 |
| Frontal_Mid_R | 28.5 | 30 | 28.5 | 2.8681 | 56 |
| Frontal_Sup_R | 13.5 | 31.5 | 48 | 2.6785 | 35 |
| Paracentral_Lobule_L | -9 | -28.5 | 69 | 2.2766 | 13 |
| Precentral_L | -49.5 | -1.5 | 40.5 | 2.5778 | 47 |
| Postcentral_L | -48 | -36 | 55.5 | 2.628 | 78 |
| Postcentral_R | 16.5 | -42 | 72 | 2.3825 | 119 |
| Caudate_L | -7.5 | 6 | 15 | 2.8678 | 593 |
| Caudate_R | 12 | -4.5 | 25.5 | 3.5166 | 371 |
| Thalamus_R | 7.5 | -19.5 | 16.5 | 3.6152 | 89 |
| Thalamus_L | -3 | -15 | 18 | 2.5933 | 30 |
| Cerebellum Anterior Lobe | -6 | -43.5 | -37.5 | 2.6109 | 133 |
| Cerebelum_6_L | -31.5 | -60 | -22.5 | 2.678 | 41 |

Abbreviations: CaSCN, causal network of structural covariance; MNI, Montreal Neurological Institute; GC, granger causality values; L, left; R, right;

Table S4. SCN alterations in NMHE and MHE relative to HCs respectively

| Brain regions | MNI coordinates (x, y, z) | | | Peak t-value | Number of voxels |
| --- | --- | --- | --- | --- | --- |
|  | x | y | z |  |  |
| NMHE vs HCs |  |  |  |  |  |
| Cerebelum_9_L | -15 | -39 | -48 | -3.5528 | 22 |
| Vermis_1_2 | -3 | -39 | -24 | -3.2652 | 18 |
| Cerebelum_Crus1_L | -25.5 | -63 | -34.5 | -2.729 | 18 |
| Frontal_Mid_R | 37.5 | 18 | 49.5 | -2.7022 | 60 |
| Frontal_Med_Orb_L | -12 | 67.5 | -12 | -3.4174 | 110 |
| Frontal_Med_Orb_R | 7.5 | 61.5 | -12 | -2.6852 | 17 |
| Frontal_Inf_Tri_L | -37.5 | 30 | 24 | -3.3343 | 94 |
| Temporal_Inf_R | -15 | 12 | -6 | -3.2414 | 111 |
| Temporal_Inf_L | -45 | 6 | -37.5 | -2.8675 | 10 |
| Temporal_Mid_R | 57 | -45 | -3 | -4.2744 | 63 |
| Occipital_Mid_L | -18 | -90 | 3 | -3.7338 | 63 |
| Supp_Motor_Area_R | 0 | -19.5 | 69 | -2.9019 | 15 |
| Precentral_R | 46.5 | 0 | 31.5 | -2.5496 | 25 |
| Precentral_L | -34.5 | 0 | 43.5 | -3.6046 | 14 |
| Calcarine_R | 13.5 | -73.5 | 13.5 | -3.2387 | 38 |
| Calcarine_L | -7.5 | -88.5 | 1.5 | -3.0266 | 46 |
| Lingual_L | 51 | -49.5 | -27 | -3.1617 | 37 |
|  |  |  |  |  |  |
| MHE vs HCs |  |  |  |  |  |
| Cerebelum_9_R | 4.5 | -36 | -61.5 | -3.9211 | 25 |
| Cerebelum_9_L | -15 | -45 | -55.5 | -3.3855 | 15 |
| Cerebelum_8_R | 21 | -61.5 | -40.5 | -2.7932 | 31 |
| Cerebelum_Crus1_R | 36 | -57 | -34.5 | -4.1357 | 32 |
| Cerebellum Anterior Lobe | 12 | -48 | -34.5 | -3.2051 | 29 |
| Dentate | -12 | -49.5 | -31.5 | -3.0503 | 103 |
| Fusiform_R | 37.5 | -69 | -16.5 | -2.782 | 10 |
| Fusiform_L | -34.5 | -52.5 | -12 | -4.1731 | 92 |
| Hippocampus_R | 16.5 | -4.5 | -10.5 | -3.2176 | 56 |
| Hippocampus_L | -16.5 | -4.5 | -6 | -3.2391 | 36 |
| Caudate_R | 12 | 7.5 | -7.5 | -3.2641 | 109 |
| Caudate_L | -7.5 | 6 | -3 | -3.4117 | 82 |
| Frontal_Mid_L | -31.5 | 22.5 | 31.5 | -3.2239 | 11 |
| Frontal_Inf_Orb_L | -48 | 16.5 | -6 | -2.7906 | 10 |
| Frontal_Sup_Orb_L | -10.5 | 67.5 | -10.5 | -3.2423 | 30 |
| Temporal_Inf_R | 48 | -12 | -27 | -2.8478 | 51 |
|  |  |  |  |  |  |
| MHE vs NMHE |  |  |  |  |  |
| Cerebelum_9_L | -19.5 | -49.5 | -43.5 | -2.8873 | 11 |
| Cerebelum_8_L | -12 | -49.5 | -31.5 | -3.0503 | 46 |
| Cerebelum_8_R | 21 | -58.5 | -39 | -2.5919 | 10 |
| Cerebelum_9_R | 10.5 | -48 | -34.5 | -3.2229 | 34 |
| Hippocampus_R | 30 | -9 | -13.5 | -3.0642 | 17 |
| Hippocampus_L | -16.5 | -4.5 | -6 | -3.2391 | 30 |
| Caudate_L | -7.5 | 6 | -3 | -3.4117 | 19 |
| Caudate_R | 9 | 10.5 | -7.5 | -3.0812 | 35 |
| Frontal_Sup_Medial_L | -13.5 | 61.5 | -4.5 | -3.3539 | 11 |
| Cingulum_Ant_L | -6 | 36 | 4.5 | -3.0685 | 25 |
| Occipital_Sup_L | -12 | -91.5 | 7.5 | -3.4584 | 18 |
| Frontal_Mid_L | -34.5 | 30 | 28.5 | -3.4228 | 17 |
| Cingulum_Mid_R | 15 | 10.5 | 40.5 | -3.9494 | 47 |
| Inter-Hemispheric | 0 | -43.5 | 69 | -3.1763 | 9 |

Abbreviations: SCN, structural covariance network; NMHE, no minimal hepatic encephalopathy; MHE, minimal hepatic encephalopathy; HCs, healthy controls; MNI, Montreal Neurological Institute; L, left; R, right;

**References**

1. von Elm, E., et al., *The Strengthening the Reporting of Observational Studies in Epidemiology (STROBE) statement: guidelines for reporting observational studies.* Lancet, 2007. **370**(9596): p. 1453-7.

2. Lv, X.F., et al., *Abnormal regional homogeneity of resting-state brain activity in patients with HBV-related cirrhosis without overt hepatic encephalopathy.* Liver Int, 2013. **33**(3): p. 375-83.

3. Ye, M., et al., *Aberrant inter-hemispheric coordination characterizes the progression of minimal hepatic encephalopathy in patients with HBV-related cirrhosis.* Neuroimage Clin, 2020. **25**: p. 102175.

4. Ashburner, J., *A fast diffeomorphic image registration algorithm.* Neuroimage, 2007. **38**(1): p. 95-113.
